# E(*n*) Equivariant Graph Neural Network for Learning Interactional Properties of Molecules

**DOI:** 10.1021/acs.jpcb.3c07304

**Published:** 2024-01-17

**Authors:** Kieran Nehil-Puleo, Co D. Quach, Nicholas C. Craven, Clare McCabe, Peter T. Cummings

**Affiliations:** † Interdisciplinary Material Science Program, Vanderbilt University, Nashville, Tennessee 37235, United States; ‡ Department of Chemical and Biomolecular Engineering, Vanderbilt University, Nashville, Tennessee 37235-1826, United States; § School of Engineering and Physical Sciences, Heriot-Watt University, Edinburgh EH14 4AS, Scotland, U.K.

## Abstract

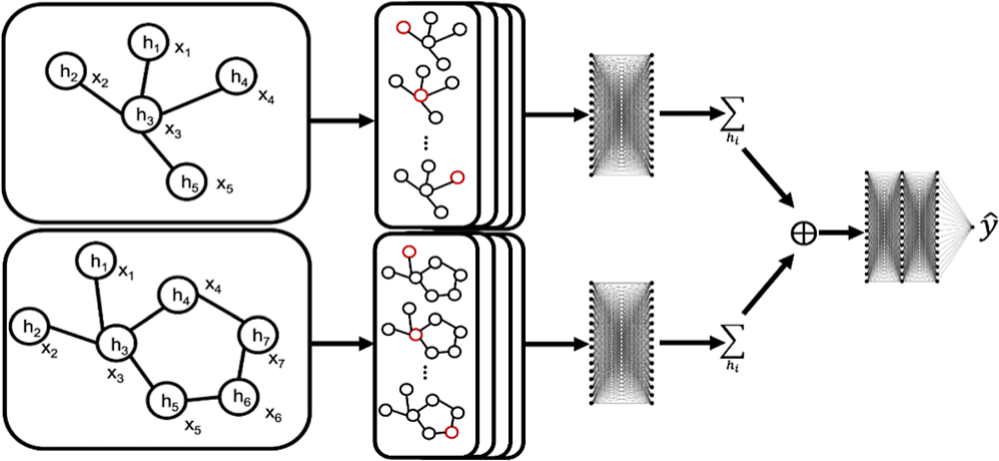

We have developed a multi-input E(*n*) equivariant graph convolution-based model designed for the prediction of chemical properties that result from the interaction of heterogeneous molecular structures. By incorporating spatial features and constraining the functions learned from these features to be equivariant to E(*n*) symmetries, the interactional-equivariant graph neural network (IEGNN) can efficiently learn from the 3D structure of multiple molecules. To verify the IEGNN’s capability to learn interactional properties, we tested the model’s performance on three molecular data sets, two of which are curated in this study and made publicly available for future interactional model benchmarking. To enable the loading of these data sets, an open-source data structure based on the PyTorch Geometric library for batch loading multigraph data points is also created. Finally, the IEGNN’s performance on a data set consisting of an unknown interactional relationship (the frictional properties resulting between monolayers with variable composition) is examined. The IEGNN model developed was found to have the lowest mean absolute percent error for the predicted tribological properties of four of the six data sets when compared to previous methods.

## Introduction

The enormous compositional space available when designing new molecules necessitates the development of efficient methods for estimating molecular properties. For example, the size of the design space for molecules of up to 17 atoms of C, N, O, and S is estimated to be 166.4 billion unique molecules.^1^ To reduce the cost of characterizing and exploring such vast chemical design spaces, quantitative structure–property relationship (QSPR) models can be used. QSPRs can efficiently give an estimate of the collective properties of a molecular system, given a quantitative description of the chemical structures of the constituent molecules. Thus, QSPRs allow for the prediction of chemical and material properties of uncharacterized molecules, greatly reducing the costs of screening a chemical library for a molecule with ideal properties.

Models that have been traditionally used for QSPR prediction include, but are not limited to, multilinear regression, polynomial regression, and random forests.^2−4^ The aforementioned models have largely been used due to their simplicity, explainability, and ease of model implementation. By explainable, we mean that the importance of features of the input on model predictions can be estimated. These models are relatively easy to implement since they are formulated from matrix multiplications, which enable parameters to be easily optimized. More recently, deep neural network-based models have gained popularity in developing QSPR relations, despite the difficulty of prediction explainability and the complex implementation required, largely due to the predictive power of this class of models. Examples of deep neural network-based models that have been used for predicting chemical properties include conventional multilayer perceptron (MLP), graph neural networks (GNNs), 3D-convolutional neural network, and variational autoencoder.^5−7^ Properties that have been predicted with deep neural networks are very diverse but include toxicity,^8^ solubility,^9^ antibiotic activity,^10^ and tribological behavior,^11−13^ among others (see for example^12^).

Predicting the tribological properties of materials is important to numerous industrial applications. Tribological properties include viscosity of bulk systems and the coefficient of friction (COF) of interfacial systems. The prediction of these properties is a nontrivial task requiring the intelligent design of models and the laborious collection of characterization data for different materials. Previous QSPR models for the prediction of tribological properties have relied on molecular descriptors and MLP-structured models.^11−13^ In these studies, the effects of various lubricants on the friction between an experimentally characterized interface and a QSPR model were built to predict the wear reduction induced by the lubricant.

Of the various classes of deep learning models that have been used to predict molecular properties, recently GNNs have gained in popularity^14,15^ due to their ability to learn directly from molecular structures. GNNs are a general class of models that can learn to perform different graph-based predictive tasks such as node inference, edge inference, and graph-level classification or regression. GNNs have been applied to a wide range of graph-based problems such as recommendation systems,^16^ chemical reaction prediction,^17^ and numerous others (see for example refs (18–20)). In the context of chemistry, GNNs learn to make predictions from the structures of molecular graphs. This type of learning is in contrast to methods that utilize intermediary representations of the molecular graph structure, such as Morgan fingerprint,^21^ and sequence-based representations, such as SMILES^22^ and SELFIES.^23^

The graph data structure consists of a set of nodes connected by edges, formally: *G* = (*N*, *E*). The superior performance of GNNs in molecular tasks is likely due to the efficient representation of molecules as graphs: atoms (represented by nodes) connected by bonds (represented by edges). This efficient representation results in a reduction in the cardinality of the set unique descriptions needed to encode a molecular structure and thus may lead to more efficient learning.^24^

There are several variations of neural network layers that can learn about graph structures such as the MPN,^25^ GraphSAGE,^26^ and GAT.^27^ The variation on which we base the model developed herein uses multiple feed-forward neural networks to learn graph convolutions.^28^

Learning in GNNs is typically performed by graph convolution layers (GCLs) which enable the learning of convolutional functions that combine structural patterns in the graph to enable information about the graph structure to be learned. By applying several GCLs in series, the GNN is able to learn to combine structural patterns in the graph to make a prediction. The GCL is defined as
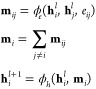
where ϕ_*e*_ and ϕ_*h*_ are feed-forward fully connected neural networks for the edge and node features, **h**_*i*_^*l*^, *e*_*ij*_, and **m**_*i*_ are the node features of node *i* after GCL layer *l*, the edge features between node *i* and *j*, and the “message” accumulated, respectively.

Conventional GNNs are permutation equivariant networks that operate on graph structured data. GNNs typically do not include the spatial positions of the nodes of a graph such as the atomic positions of a molecule in space. Recently GCLs have been constrained to be equivariant in the handling of spatial features.^15^ Equivariance to the Euclidean group [E(*n*)] was applied to create an equivariant graph convolution layer (EGCL) which enabled the construction of an equivariant graph neural network (EGNN) models.^15^ The EGNN’s major innovations were the utilization of spatial features for predictions and the sensible treatment of transformations to these features. The EGNN showed significant improvement in prediction accuracy as well as a reduction in the number of training examples needed to learn tasks. These constraints enabled the learning of transformations on the spatial coordinates that are equivariant with the symmetries of the Euclidean group. More specifically, equivariance on the Euclidean group has been imposed on graph-based models to enable the learning of transformations of spatial features that are equivariant to rotations and translations, and therefore reflections as well.

As stated previously, GNNs learn information about graphs *G* = (*N*, *E*), where spatial features may be included in the node features of the graph, but the spatial features would be treated the same as other node features, meaning without E(*n*) equivariance. Unlike GNNs, EGNNs treat the coordinates as a separate category of information, *G* = (*N*, *E*, *X*). Concisely, the EGCL performs a transformation on node and coordinate features, **h**^*l*+1^, **x**^*l*+1^ = EGCL(**h**^*l*^, **x**^*l*^, *e*_*ij*_) where **h**^*l*+1^ are the node features, **x**^*l*^ are the node coordinate embeddings, *e*_*ij*_ are the edge features between node *i* and *j*, after layer *l* convolution, ϕ_*e*_, ϕ_*x*_, and ϕ_*h*_ are MLP model used for the edges, coordinates, and node features, respectively. In detail, each EGCL performs
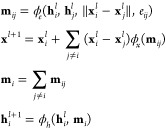


Intuitively, the EGCL must have the property that,
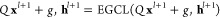
where *Q* is a rotational or reflectional transformation and *g* is a translational transformation s.t. *Q* ∈ ^d*d^, and *g* ∈ ^*d*^, where *d* is the dimensionality of the coordinate space of **x**.

Equivariant mapping, or equivariance, is a constraint placed on transformations that arise from physical considerations of the group.^29^ In particular, equivariance is used to describe the symmetry of operations on groups. Equivariance enables models to produce equally varying predictions. In simple terms, this means that our model should learn the properties of graph structures regardless of whether they are reflected, translated, or rotated in space. By constraining the model to be equivariant on the Euclidean group, the input space needed to be learned is significantly reduced, thereby making the model more robust and efficient at learning from molecular data. In theoretical descriptions of molecular funds, invariance of the interaction potential between molecules with respect to translation, rotation, and reflection of the molecule pair yields a powerful reduction in the dimensionality of the representation of the interaction (as well as relative structure) of the molecules that is needed in theoretical descriptions of molecular fluids using statistical mechanics.^30−33^

Formally, equivariance is defined^15^ as follows. Let *T*_g_: *X* → *X* be a set of transformations on X for the group *g* ∈ *G*. A function ϕ: *X* → *Y* is equivariant to *g* if there exists an equivariant transformation on its output space *S*_*g*_: *Y* → *Y* such that ϕ(*T*_g_(*X*)) = *S*_*g*_(ϕ(*X*)).

A problem not commonly addressed in QSPR models is the fact that many of the chemical properties these models try to predict result from the interaction of multiple dissimilar molecules. Examples of interactional properties are the COF, which is conditional on both molecules rubbing against each other, and the binding energy of a drug, which is dependent on the structure to which it is binding to. In most current QSPR models, the interactional nature of the data is ignored, and predictions are made by looking at the properties of just one of the molecules. This is a major limitation for predicting properties that result from the interaction of multiple molecular species. Modifications previously made to GNNs to enable predictions from multiple input graphs are spatio-temporal graph convolution networks,^34^ graph co-attention networks,^35^ and Multi-Resolution GNN.^36^ Unfortunately, models like spatio-temporal graph convolution networks rely on temporal layers between the nodes in the input graphs, necessitating input graphs to possess the same number of nodes. Models such as graph co-attention rely on a variation of the attention mechanism^37^ to enable “attention” to be paid to the interaction between atoms in the molecule. The effects of the addition of a linear attention layer to the GCL was examined by the authors, but no improvement in the performance was observed. Due to the promise of EGNNs and multi-input graph-based models to learn molecular properties, we seek to further extend the conventional single-input EGNN to learn properties that result from the interaction of multiple atomic graphs in 3D space.

## Methods

### Data Sets

#### Simplified

For an initial test of the performance of the models examined in this study, a simplified interactional data set was created that used the total number of atoms resulting from the molecule pair. For the graph-based models used for this task, the node features were the one-hot encoding of the atom type. One-hot encoding is a method for the conversion of categorical data into a format that can be input into machine learning (ML) algorithms. The node and coordinate features were extracted from the SMILES representations of the molecules using the mBuild^38^ hierarchical, component-based molecular system building package. The edge features were not used for this task.

To generate the data set, pairs of molecules were selected from a random uniform distribution of the GDB chemical universe data set.^1^ The GDB data set was selected to ensure that the structures of the selected molecules possessed no bias toward a particular structure and therefore preserved the generality of this benchmarking data set.

#### SASA

For an interactional property of intermediate difficultly that results from 3D spatial properties, the solvent accessible surface area (SASA) was used. The SASA is calculated using a sphere of a radius approximating the solvent molecule to “probe” the surface of the solute molecule by rolling it along the spheres of the solute molecule. The SASA is an intermediate task for an interactional model to predict because the approximate radius of the solvent molecule must be estimated as well as the accessible surface of the solute molecule. This means that a model that appropriately learns to predict the SASA requires information from the spatial features of both molecules.

The selection of molecule pairs and the preparation of node and coordinate features were performed in the same way as for the simplified data set. To calculate the resultant SASA the RDKit,^39^ chemoinformatics package was used. The RDKit package utilizes the FreeSASA^40^ algorithm to calculate the SASA. For this task, the node features were the one-hot encoding of the atom type and the van der Waals radius of the atom. The edge features were not used for this task.

#### Lubricating Thin Films

Monolayer film coatings have shown potential as a means of lubricating surfaces with micro- and nanometer separations. Such layers of coatings could provide protection to the surfaces and minimize frictional forces that incur when these surfaces come into contact. An optimized coating could remove design constraints as well as increase the stability and lifetime of micro- and nanoscale systems/devices. Monolayer films are composed of a layer of grass-like molecules, where each molecule is made up of a headgroup, a backbone or space chain, and a terminal group. Each component of the molecules has been shown to impact the lubricating ability of the film, though, the terminal group has been shown to have the largest effect.^3,41−45^ Multicomponent monolayer films, where each monolayer is made up of two or more types of terminal group can also affect the lubricating ability.^3,42,44,46^ This presents a huge parameter space to be investigated, making it impossible to exhaustively study such systems via experiments or computational simulations. Hence, having an effective QSPR model to extrapolate and predict the lubricating efficacy of different monolayer film coatings is crucial to designing optimal lubricating films. The lubricating properties of monolayers are examples of interactional properties with unknown functional forms, representing an increased level of complexity compared to the problem presented in the simplified and SASA data sets.

**Figure 1:**
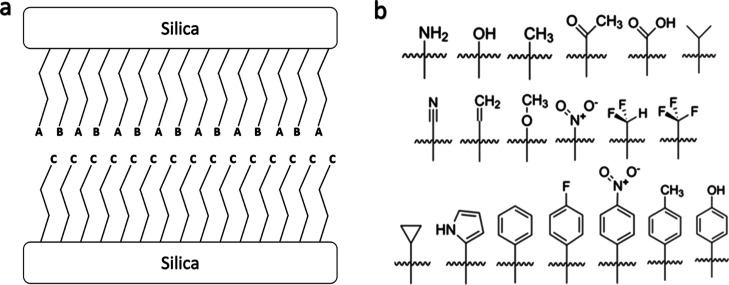
(a) Simplified schematic of the systems studied. The top monolayer is a mixture of two types of terminal group chemistries (A,B), studied at two different mixing ratios (25:75 and 50:50), while the bottom monolayer is homogeneous (chemistry C). (b) Depiction of the 19 different chemistries considered for alkylsilane end groups. From top to bottom, left to right, the terminal groups are amino, hydroxyl, methyl, acetyl, carboxyl, isopropyl, cyano, ethylene, methoxy, nitro, difluoromethyl, perfluoromethyl, cyclopropyl, pyrrole, phenyl, fluorophenyl, nitrophenyl, toluene, and phenol.

In previous studies from our group, the tribological properties of different monolayer films were investigated using nonequilibrium molecular dynamics simulations.^3,41,47^ In this paper, we are utilizing the data set reported in studies by Summers et al.^41^ and Quach et al.^3^ A schematic of the systems is shown in Figure 1. Both studies focused on the effect of the terminal group on the lubricating properties of the thin monolayer film, though the Quach et al.^3^ study also considered the effect of multicomponent monolayers, i.e., thin films composed of two or more terminal groups at different compositions. The Summers et al.^41^ study investigated 100 unique systems, while Quach et al.^3^ considered 9672 unique system designs, together creating a pool of 9772 data points.

In the Quach et al.^3^ study, the tribological data set was used to train a random forest ML model. Each system is represented by its molecular “fingerprint”, which is a combination of molecular descriptors for component terminal group chemistries. Molecular descriptors represent physical and chemical properties of molecular chemistry that can be used for QSPR analysis and can be divided into four categories: size, shape, complexity, and charge distribution. The molecular descriptors of each terminal group were calculated with RDKit, through its corresponding hydrogen- and methyl-capped structure. The hydrogen-capped structure was used to determine properties relating to shape, and the methyl-capped structure was utilized to calculate the remaining properties. The hydrogen-terminus structure was found to be sufficient in describing shape-related properties, and the methyl-terminus structure was found to better approximate other properties assimilating scenarios when the terminal group is attached to the alkyl chain. Each of these structures were represented by 53 descriptors, summarized in the Supporting Information (see Table S1). Through this procedure, the molecular descriptors for the top and bottom monolayers are first independently calculated. The descriptors of heterogeneous monolayers, i.e., monolayers made up of two terminal groups, are weighted averages (by relative composition) of the components of terminal group descriptors. Descriptors of the two monolayers are combined, storing their mean and minimum for each pair of descriptors, forming the raw “fingerprint” of the system. Even though this representation does not fully capture the complete monolayer structure, such as information related to the distribution/clustering of chains in the monolayer, because we are primarily interested in the effect of terminal group chemistries on the tribological properties of the film coatings, such a set up was found to be sufficient. These values underwent further feature reduction steps through which those that had low variance or were highly correlated were removed and reduced. Descriptors whose variance were less than 2% were removed, while groups with greater than or equal to 90% correlated were reduced to a single attribute. The reduced list of molecular descriptors is then used as the input parameters to the ML models.

**Figure 2:**
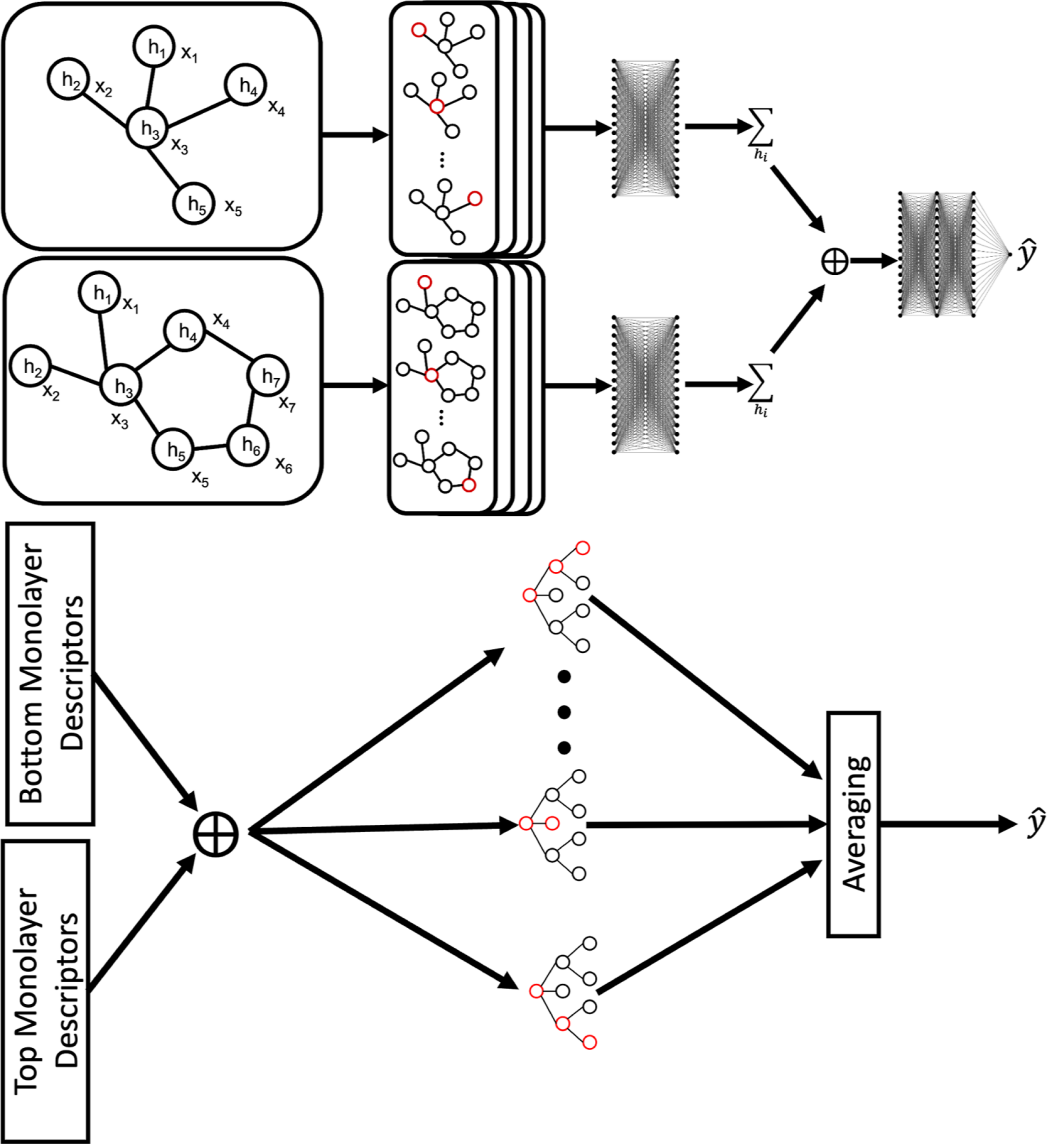
(Top model) Schematic of the IEGNN model architecture. (Top far left) Input graphs can have different number of nodes, where *h*_*n*_ are node features and *x*_*n*_ are coordinate features for node *n*. (Top second from left) Multiple EGCL are used to learn the information about the graph structure. (Top right) Information is learned in both graphs and the information is accumulated across each graph separately and is finally concatenated and fed into another MLP to make a final prediction. (Bottom model) Schematic of the random forest model used in ref (3). Final prediction output is made by averaging across the predictions made by the ensemble of decision trees. One of the main differences between the two models is additional alterations to monolayer descriptors that occur prior to concatenation in the IEGNN.

In summary, Quach et al.^3^ performed a large-scale molecular dynamics screening study to produce a data set consisting of the frictional properties (COF, and the adhesive force, *F*_0_, that measures the force required to overcome the attraction between the two functionalized surfaces, see Figure 1) that resulted from the shearing of monolayers with of alkylsilane chains with different terminal groups. Quach et al.^3^ then used an ensemble of decision trees (a random forest) to predict the tribological properties of various monolayers using the monolayer descriptors described above (see Figure 2 for a depiction of the model).

For models reported herein, in addition to the molecular descriptors obtained through RDKit, created through the procedure described above, we also included coordinate information on the end groups. We loaded the 3D molecule structures from their SMILES representation using mBuild and then processed these structures into bond edge lists, coordinates, and node features for the graph-based models. To get these 3D molecular structures from SMILES we used mBuild since, in our experience, it tends to produce more physically realistic 3D structures than RDKit. For the graph-based models, the node features were the one-hot encoding of the atom type concatenated with the previously used molecular descriptors described above. For the purely MLP-based models, the monolayer descriptors outlined in the preceding paragraph were the only inputs.

### Multi-Graph Data Point

Due to the limitations of working memory, the entire data set cannot be loaded at once and must be loaded in batches. Batch loading presents difficulties because batch loading of graph data requires special handling of the data that is distinct from the image or sequence data. To enable batch loading of multigraph data, we created a custom data structure based on the PyTorch Geometric^48^ (PyG) data set.

To create the custom PyG data set, we subclassed the PyG.Data object and made a modification to allow for data points to consist of a variable number of graphs. Multigraph data points can be stored in a python list for further processing by PyG’s collate function. The PyG collate function conglomerates all multigraph data points into a PyG data set which can then be batch loaded. Because saving a large python list of graph structures is inefficient, PyG collates the list into one very large PyG Data object before it is saved to memory. The collated data object concatenated has all examples combined into one big data object and, in addition, returns a slices dictionary to reconstruct single examples from this object.^48^ To the authors’ knowledge, this is the first openly accessible implementation of a PyG data set that can batch load any number of input graphs for a data point. This data structure is open-access and can be used by installing the mData python software package∥ data structure for the loading of multigraph data points. Example usage for creating a data point is shown in Listing 1.

Listing 1: Example usage of the mData multi-graph input data structure.
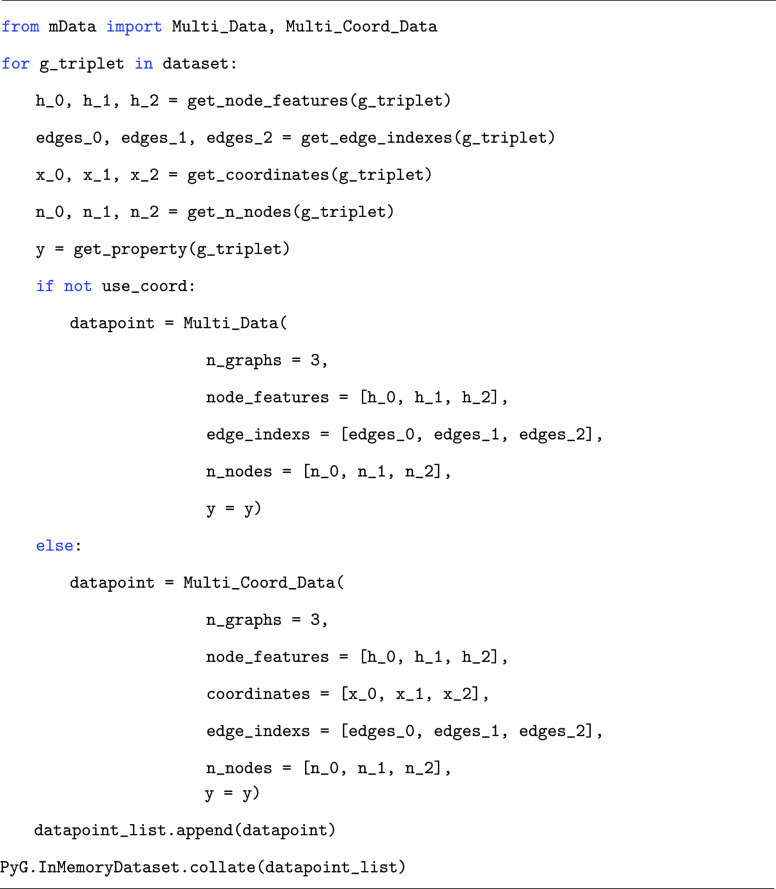


### Interactional E(*n*) Graph Neural Network

Previous models for the prediction of tribological properties do not incorporate multiple graph inputs and were therefore unable to learn information that results from the interaction of these different molecules. To address this shortcoming, we created a model that learns structural information from each graph input and then learns how to combine this information to understand the interaction of the input graph structures. We call this model the interactional-equivariant graph neural network (IEGNN). We created this model using the ML framework PyTorch.^49^ The IEGNN is composed of EGCL, MLPs, vector concatenation, and a node-level summation. The structure of the IEGNN can be broken down into four components. The first is a series of ECLs that accumulate information about the molecular structure of the molecules [Figure 2 (top)]. The next component is a MLP, this layer transforms the features learned from structural information learned from the ECLs into a latent form. The third layer consists of a global pooling layer which sums the node values of the transformed graph together to create a global, or overall, molecular descriptor. The final layer is another MLP that transforms the global molecular descriptor to make the final property prediction. The algorithm for whole IEGNN is also detailed below (see Algorithm 1).
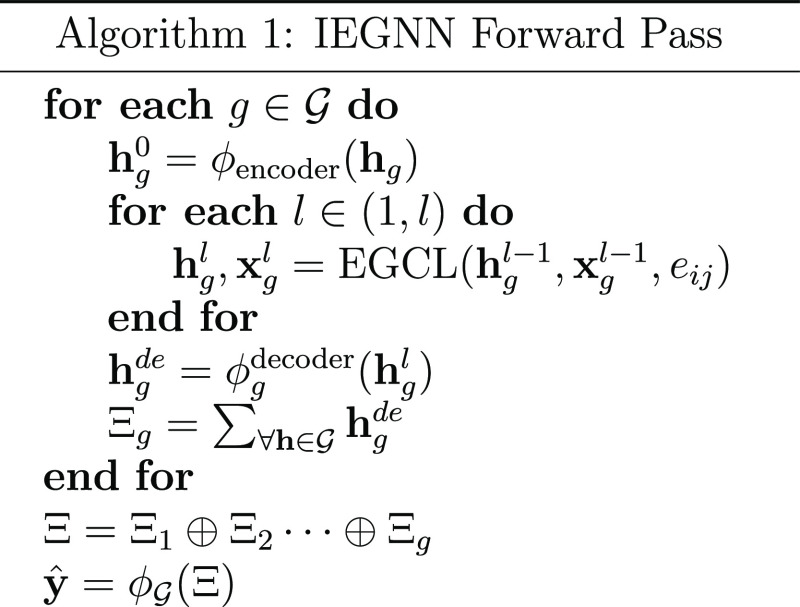
where  is the set of input graphs, **h** are node features, *l* is the number of layers, **x** are the coordinate features, *e*_*ij*_ is the edge between node *i* and node *j*, and ϕ represents a MLP⊥.

### Training Workflow

**Figure 3:**
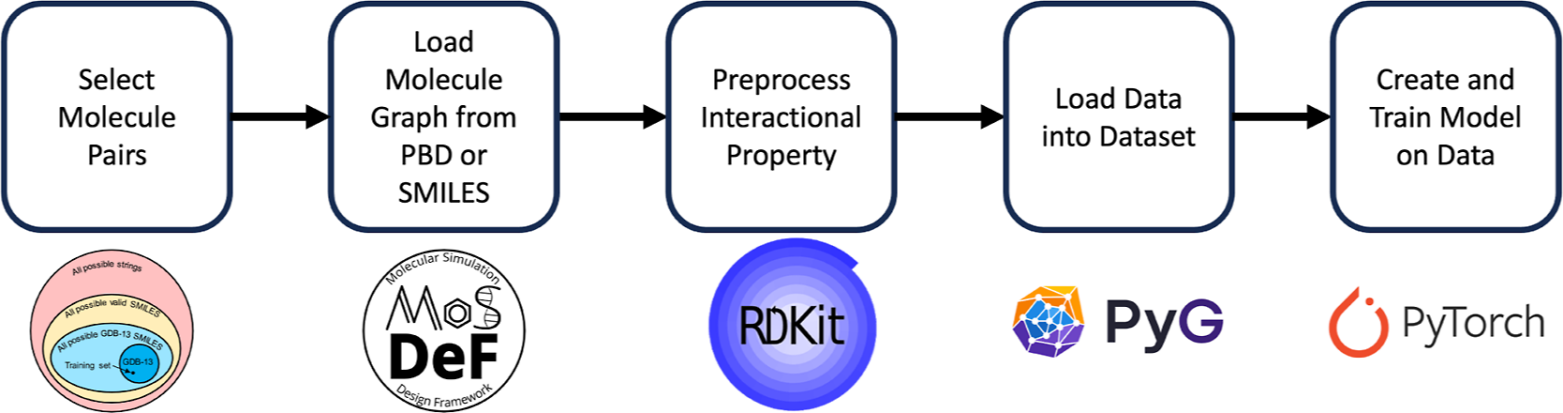
Computational workflow and respective software packages used to process the data set, load the data, and train the model.

The workflow used for training the models from the SASA and simplified data sets can be described in five steps (see Figure 3 for visualization and associated software needed for each step):

1. Select two molecules randomly from the GDB SMILES library.
2. Load the molecular graph structure and estimates of the atomic coordinates from the mBuild package.
3. Estimate the interactional property either from RDKit or a simple arithmetic calculation.
4. Load the data into the mData data structure and collate all data points together using the PyG data set data structure.
5. Define the model architecture using PyTorch and load the data points in batches from the data set for model training

This workflow is nearly identical to the method used for the monolayers data set with differences only in steps 1 and 3; therefore, the workflow for the monolayers data set was not discussed.

## Results and Discussion

With a 80–20 training-testing split, the IEGNN possessed a mean absolute percent error (MAPE) approximately 5% smaller than the random forest model previously used for predicting the tribological properties of monolayers (see Table 1 for a summary of performance results and Figure 2 for a comparison of model architecture).

**Table 1:** Overview of the Model Performance on Test Set for the Various Data Sets Described^a^

data set	random forest^3^	IEGNN	IGNN	IMLP
simple	–	0.000106	0.0661	–
SASA	–	0.0362	0.0440	–
monolayers (50:50) (COF)	**0.0196**	0.0389	0.0582	0.0807
monolayers (25:75) (COF)	0.0505	**0.0460**	0.0694	0.0881
monolayers (all) (COF)	**0.0230**	0.0308	0.0601	0.0855
monolayers (50:50) (*F*_0_)	0.150	**0.141**	0.396	0.630
monolayers (25:75) (*F*_0_)	0.245	**0.198**	0.378	0.773
monolayers (all) (*F*_0_)	0.179	**0.165**	0.368	0.624

a Model performance is measured in MAPE. Comparison of the test set performance to previous models on the monolayer, with the best-performing bolded.

Unsurprisingly, the IEGNN and IGNN were able to achieve an extremely small MAPE of 0.000106 and 0.0661 on the simple data set, meaning that the models were effectively learning basic interactional properties of materials, both with and without coordinate features. This is an unsurprising result since the graph structures supplied as inputs to both models contain the number of atoms implicitly by the number of nodes in the graph.

The IEGNN was able to estimate the SASA with 82.3% of the MAPE compared to the IGNN. This result is unsurprising because the SASA is highly dependent on the conformation of the molecule, which can only be described by the coordinate features of the molecule.

In addition to the graph-based models, we compared the performance of a nongraph-based model, the interactional MLP (IMLP), to determine the effects of including the molecular graph as inputs. The inclusion of the graph structure as the input for the prediction of *F*_0_ resulted in 22.3 and 62.9% of the MAPE for the IEGNN and IGNN, respectively, when compared to the IMLP model. This reduction in MAPE with the addition of graph structures demonstrated the importance of including the molecular graph for the development of QSPRs.

More accurate QSPR models would lead to improved screening surveys of molecules, which, in turn, would reduce the cost of material or chemical selection. In addition, the IEGNN’s separate treatment of molecule inputs enables the screening of molecule pairs that are conditional on one of the molecules. This ability would enable the selection of materials given the known presence of another molecule. If it is verified that the model learns information about each graph independently, then it would mean that different molecular interactions of the same molecule could be used to infer information about other interactions.

### Random Forest Compared to the Interactional E(*n*) Graph Neural Network

**Figure 4:**
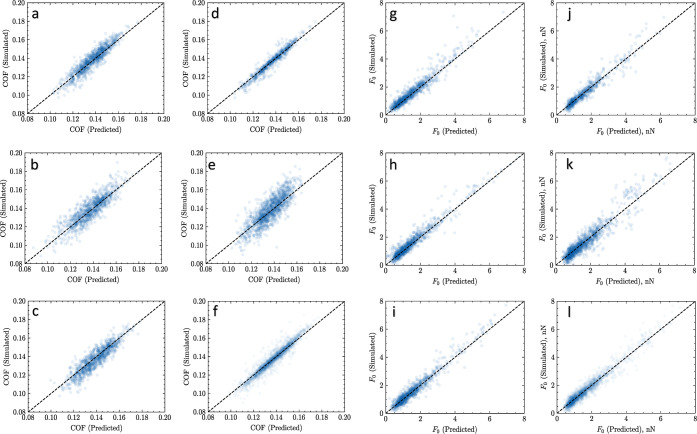
Predicted vs simulated tribological values (COF and *F*_0_) for the herein developed IEGNN model and the previously developed random forest model. All values were taken at random from the training sets. The dashed diagonal line corresponds to a perfect prediction, meaning the greater the deviation from the line, the greater the error. The two columns (a–c,g–i) correspond to predictions made with the IEGNN model, whereas the other two columns (d–f,j–l) correspond to predictions made with the random forest model. The row (a,d,g,j) corresponds to training using the 50:50 data subset. The row (b,e,h,k) corresponds to the data 25:75 subset. The row (c,f,i,l) corresponds to the full training data set. The first two columns (a–f) are for the COF, and the last two columns (g–l) are for the *F*_0_.

The primary difference between the random forest model and the IEGNN is the inclusion of the raw spatial/atomic and bond features. The random forest is reliant on an encoding of this information into a vectorized representation, which may limit the patterns between data points that can be learned. The IEGNN is found to outperform the tribological prediction of the previous model for four of six data sets (see Figure 4 for a visualization of performance and Table 1 for quantitative comparison of performance). Notably the IEGNN consistently outperforms the previous random forest model in the prediction of *F*_0_ but not the COF. This suggests that *F*_0_ is more dependent on the spatial and geometrical features of the molecule since this is the major difference between the random forest and the graph-based models. Since *F*_0_ is a result of the attractive forces between the molecules, as shown in the work of Summers et al.,^41^ this property may be more easily derived purely from the chemical structure. The COF however is a transport property, more aligned with the shape of the terminal groups.^41^ It therefore may be more difficult for a model to capture shape information without additional training and/or data.

### Inclusion of E(*n*) Features

**Figure 5:**
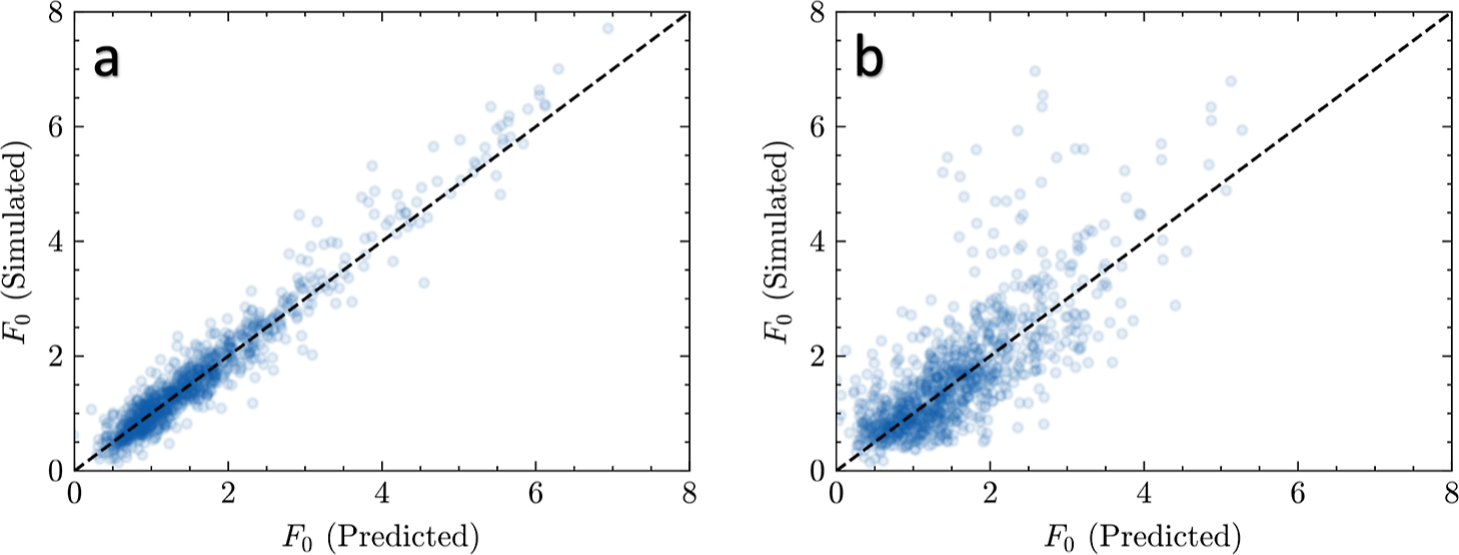
(a) IEGNN prediction of COF from the monolayer screening data set. (b) IGNN prediction of COF from monolayer data set. This plot shows the IGNN’s inaccuracy in predicting values with lower *F*_0_ in the data set and the importance of including spatial features when making QSPR predictions.

To gain a deeper understanding of the impact of coordinate features on the model’s accuracy, an experiment was conducted where a separate model with the identical architecture as the IEGNN was trained, but this time, without incorporating the atomic coordinates (see Figure 5). With the inclusion of coordinate features, the model was able to make predictions for the *F*_0_ of a 50:50 monolayer system with 35.6% the error of the same model without atomic coordinates. These results suggest that the 3D spatial features are important for the prediction of tribological properties. This result also suggests that the IEGNN is able to learn spatial features not explicitly included in the molecular “fingerprint”.

### Inclusion of Graph Structures

To gain a deeper understanding of the impact of the inclusion of molecular graph information on the model’s accuracy, a separate model was trained with the same vector concatenation of features between end groups but this time using a MLP, called the IMLP, as the molecular encoder. With the inclusion of graph structural information, the model was able to make predictions of the COF of a *F*_0_ monolayer system with 22.4% the error of the same model without the molecular graph structure. These results suggest that the use of graph-based models are important for the accurate prediction of tribological properties.

## Conclusions

We developed an E(*n*) equivariant graph convolution-based ML model, IEGNN, for the prediction of properties that result from the interaction of multiple molecular structures. To benchmark the model’s ability to predict interactional properties, we curated data for three different interactional properties, with varying degrees of interactional complexity. While for the first two data sets, the connection between the property and structure is more clear, the final data set consisted of an unknown interactional relationship, namely, the frictional properties resulting between monolayers of variable compositions. The IEGNN model developed was found to have the lowest MAPE for four of six of the tribological data sets considered, when compared to previous methods applied to the same data. We determined the performance of the model with and without spatial features and graph-based ML models, with the conclusion that the inclusion of spatial features and molecular graph features significantly improves property prediction (see Table 1 for details). To load the inputs needed for the multi-input graph model, we implemented a PyG data set using a custom data point data structure that we made publicly available for further model development. To enable the loading of these data sets, we also created an open-source data structure based on the PyG library for batch loading of multigraph data points. Finally, we created the IEGNN to estimate the interactional properties from the data sets. The creation of the IEGNN architecture enables a new equivariant graph-based method for property prediction that can be used to learn properties that result from interactional information for different molecules. By incorporating spatial features and constraining the functions learned from these features to be equivariant to E(*n*) symmetries, the IEGNN was able to efficiently learn from 3D molecular structures of multiple molecules.

## Supporting Information Available

Molecular descriptors calculated used to describe the terminal groups in the tribology model (PDF)

## Footnotes

∥ https://github.com/kierannp/mData.

⊥ The code for the IEGNN model and the custom multigraph input PyG data set can be found at https://github.com/kierannp/IEGNN.
