# Supplementary material for: E(n) Equivariant Graph Neural Network for Learning Interactional Properties of Molecules
Source: J Phys Chem B. 2024 Jan 17;128(4):1108–17. doi: 10.1021/acs.jpcb.3c07304 (PMC10839827; doi:10.1021/acs.jpcb.3c07304)
Supplement: Supplementary file 1 — jp3c07304_si_001.pdf [file jp3c07304_si_001.pdf]

Table S1. The molecular descriptors calculated from RDKit that were used to describe the terminal groups.

Molecular Descriptor	Description	Category
Approximate Surface Area	Approximation of molecular surface area using the approach defined by Labute[1]	Size
Asphericity	Measure of molecular shape (from Baumgartner[2]); *A* = 0 for spherical shape, *A* = 1 for highly prolate shapes, and *A* = 0:25 for oblate shapes	Shape
Balaban J	Related to connectivity, degree of branching	Complexity
Bertz Ct	Measure of molecular complexity through connectivity	Complexity
Chi0, Chi1	Connectivity indices	Complexity
Chi0n - Chi4n	Connectivity indices over various molecular fragments (0=atoms, 1=one bond fragments, 2=two bond fragments, etc.)	Complexity
Chi0v - Chi4v	Valence connectivity indices (0=atoms, 1=one bond fragments, 2=two bond fragments, etc.)	Complexity
Eccentricity	Shape descriptor calculated from the inertia matrix (0=spherical, 1=linear), from Arteca[3]	Shape
Hall-Kier alpha	Modifying term for kappa descriptors, related to shape/flexibility	Shape
Hall-Kier kappa1	Alpha-modified topological shape descriptor; related to complexity/number of cycles (rings) in the bond graph	Shape
Hall-Kier kappa2	Alpha-modified topological shape descriptor; related to degree of star-like bond graph vs. linearity	Shape
Hall-Kier kappa3	Alpha-modified topological shape descriptor; related to "centrality" of branching	Shape
Hydrogen bond factor	Developed in Summers *et al.*[4] work; related to ability for formation of inter-monolayer hydrogen bonds	Charge distribution/ Misc.
IPC	Complexity/connectivity descriptor estimated from adjacency matrix of bond graph	Complexity
Inertial shape factor	Characterization of molecular shape from principal moments of inertia (pm2/(pm1 *pm3), where pm1-3 are the three principal moments), from Todeschini and Consoni[5]	Shape
logP	Octanol - water partition coefficient estimated through the method of Wildman and Crippen[6] measure of hydrophobicity	Charge distribution/ Misc.
Molar refractivity	Estimation of molecular polarizability; calculated through the method of Wildman and Crippen[6]	Size
Molecular weight	-	Size
Molecular weight (heavy atoms)	Molecular weight excluding hydrogens	Size
Normalized principal moments ratios (NPR1, NPR2)	Used to characterize molecular shape, from Sauer and Schwarz[7]	Shape

Number of heavy atoms	Number of non-hydrogen atoms	Size
Number of rotatable bonds	-	Size/Shape
Number of valence electrons	-	Size
Plane of best fit	Measure of molecular planarity (0=planar, increasing with less planarity)	Shape
Principal moments of inertia (PMI1, PMI2, PMI3)	Three principal moments of inertia for the molecule (1=smallest, 3=largest)	Shape
Radius of gyration	Characterizes molecular shape, specifically, elongation	Shape/Size
Sphericity	Measure of molecular shape (0=spherical, 1=flat), from Robinson[8]	Shape
Topological polar surface area	Estimation of surface area of only polar atoms, from Ertl[9]	Charge distribution
Total hydrophobic VSA	Sum of SA contributions from atoms with 0:20 q < 0:20	Charge distribution
Total negative van der Waals sur- face area (VSA)	Sum of SA contributions from atoms with q< 0.0	Charge distribution
Total negative polar VSA	Sum of SA contributions from atoms with q< 0.2	Charge distribution
Total polar VSA	Sum of SA contributions from atoms with abs(q) > .2	Charge distribution
Total positive VSA	Sum of SA contributions from atoms with q > 0.0	Charge distribution
Total positive polar VSA	Sum of SA contributions from atoms with q>= 0.2	Charge distribution
Fractional hydrophobic VSA	Total hydrophobic VSA / Total VSA	Charge distribution
Fractional negative VSA	Total negative VSA / Total VSA	Charge distribution
Fractional negative polar VSA	Total negative polar VSA / Total VSA	Charge distribution
Fractional polar VSA	Total polar VSA / Total VSA	Charge distribution
Fractional positive VSA	Total positive VSA / Total VSA	Charge distribution
Fractional positive polar VSA	Total positive polar VSA / Total VSA	Charge distribution

References

1. Labute, Paul. A widely applicable set of descriptors. *Journal of Molecular Graphics and Modelling*, **2000**, *18, 4–5, 464–477*
2. Baumgärtner, A. Shapes of flexible vesicles at constant volume. *The Journal of Chemical Physics*, **1993**, *98, 9, 7496–7501*
3. Arteca, G. Molecular shape descriptors. *Reviews in Computational Chemistry*, **1996**, *191–253*

4. Summers, A. Z.; Gilmer, J. B.; Iacovella, C. R.; Cummings, P. T.; McCabe, C. MoS-DeF, a Python framework enabling large-scale computational screening of soft matter: Application to chemistry-property relationships in lubricating monolayer films. *Journal of Chemical Theory and Computation* **2020**, 16, 1779–1793.
5. Todeschini, R.; Consonni, V. Descriptors from molecular geometry. *Handbook of Chemoinformatics*, **2003**, 1004–1033
6. Wildman, S. A., and Crippen, G.M. Prediction of physicochemical parameters by atomic contributions. *Journal of Chemical Information and Computer Sciences*, 39, 5, **1999**, 868–873
7. Sauer, W. H.; Schwarz, M.K. Molecular shape diversity of combinatorial libraries: a prerequisite for broad bioactivity. *Journal of Chemical Information and Computer Sciences*, **2003**, 43, 3, 987–1003
8. Robinson, D.; Barlow, T.; W. Graham, R. Reduced dimensional representations of molecular structure. *Journal of Chemical Information and Computer Sciences*, vol. 37, no. 5, **1997**, pp. 939–942,
9. Ertl, P.; Rohde, B.; Selzer, P.; Fast calculation of molecular polar surface area as a sum of fragment-based contributions and its application to the prediction of drug transport properties. *Journal of Medicinal Chemistry*, **2000**, *43, 20, 3714–3717*
